# Disseminated cryptococcosis mimicking malignant lymphoma on ^18^F-FDG PET/CT: A case report

**DOI:** 10.1097/MD.0000000000031374

**Published:** 2022-10-28

**Authors:** Xinchao Zhang, Yujing Hu, Congna Tian, Qiang Wei, Yanzhu Bian

**Affiliations:** a Department of Nuclear Medicine, Hebei General Hospital, Shijiazhuang, China.

**Keywords:** cryptococcosis, *Cryptococcus gattii*, *Cryptococcus neoformans*, FDG, lymphoma, PET/CT

## Abstract

Disseminated cryptococcosis is extremely rare and is easily misdiagnosed as a malignant lymphoma. ^18^F-Fluorodeoxyglucose Positron Emission Tomography (PET)/ computed tomography (CT) may be useful to assess the involvement of disseminated cryptococcosis and to evaluate residual disease after treatment.

**Patient Concerns:**

A 21-years-old man presented with fever and cough for a month, with multiple red nodules scattered on the skin. ^18^F- Fluorodeoxyglucose PET/CT revealed multiple hypermetabolic lymph nodes in the upper and lower parts of the diaphragmatic region and hypermetabolic nodules in the skin. According to the PET/CT results, malignant lymphoma was considered a possibility, especially T-cell lymphoma involving the skin.

**Diagnosis:**

Cryptococcosis was diagnosed using inguinal lymph node biopsy and blood culture.

**Interventions:**

The patient received two months of amphotericin B, fluconazole, and half a month of meropenem.

**Outcomes:**

The patient’s body temperature returned to normal and the red nodules on the skin disappeared. Most of the hypermetabolic enlarged lymph nodes disappeared, which was confirmed by reexamination with PET/CT.

**Lessons:**

Disseminated cryptococcosis is easily misdiagnosed as malignant lymphoma, especially when the lymph nodes are more involved. When multiple hypermetabolic enlarged lymph nodes appear on PET/CT, except for lymphoma, specific infections should also be considered.

## 1. Introduction

*Cryptococcus* is an opportunistic pathogenic parasitic yeast fungus that mainly exists in animal feces, especially pigeon feces. The main pathogens in China are *Cryptococcus neoformans* (*C. neoformans*) and *Cryptococcus gattii* (*C. gattii*), with *C. neoformans* infection being the main disease in China. *C. gattii* was previously regarded as being primarily limited to tropical and subtropical regions, with a major endemic focus in Australia. It has now been described worldwide, with its emergence in Vancouver Island, British Columbia, Canada, and the U.S.^[1]^ In general, low immunity, long-term immunosuppressant use, organ transplantation, and high-dose steroid use are common risk factors for cryptococcosis. However, in recent years, the proportion of cryptococcal infections in people with normal immune function has gradually increased.^[2]^ The nervous system, lungs, and skin are the systems most easily invaded by *Cryptococcus*; therefore, most patients present with these 3 systems as the primary symptoms. There are few reports of cryptococcal lymphadenitis and here we report a case of disseminated cryptococcosis mimicking malignant lymphoma with predominantly lymph node, lung, and skin involvement.

## 2. Case report

**Figure 1.**
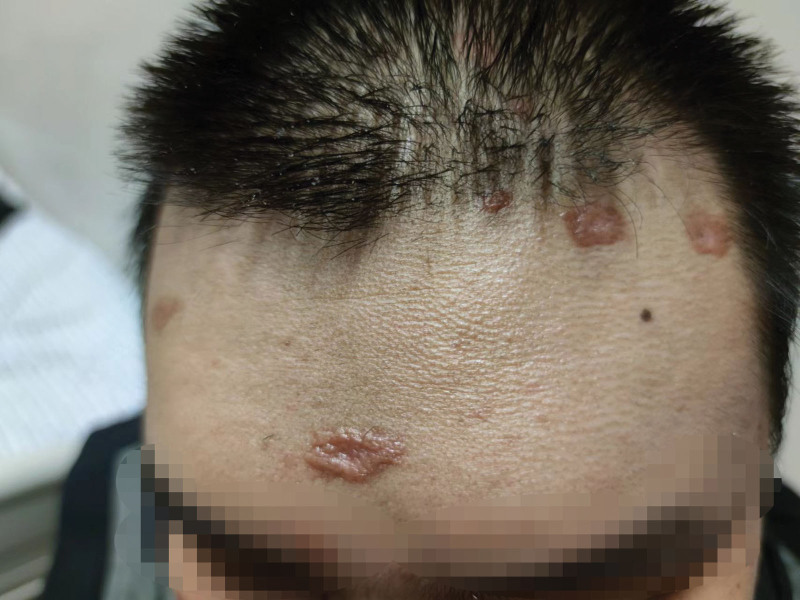
Multiple red rashes on the forehead and scalp.

A 21-years-old young college student with normal immune function had been suffering from fever of unknown origin and cough for a month with red nodules scattered on the maxillofacial skin. Her physical findings were as follows: body temperature, 37.8°C; pulse, 128 beats/minutes; and blood pressure, 169/88 mm Hg. Red papules and nodules of varying sizes were found on the head, face, and upper limbs, and some rashes were also scabbed (Fig. 1). Routine laboratory blood examination showed a white blood cell count (18.39–21.09) × 10^9^/L, eosinophil percentage (30.3–36.9) %, and eosinophil count (6.39–6.79) × 10^9^/L. Liver function tests suggested alanine aminotransferase 163.8U/L, aspartate aminotransferase 60.3U/L. Chest computed tomography (CT) showed diffuse panbronchiolitis in both lungs, and blood culture showed a coccus infection. Considering that the patient had a bloodstream infection, after consulting the pharmacy department, we decided to administer norvancomycin combined with piperacillin sodium and tazobactam sodium for anti-infection and provided the patient with glycyrrhizin for liver protection. The patient still had fever after one week of anti-infective treatment, and the inflammatory indices did not decrease significantly compared with before.

**Figure 2.**
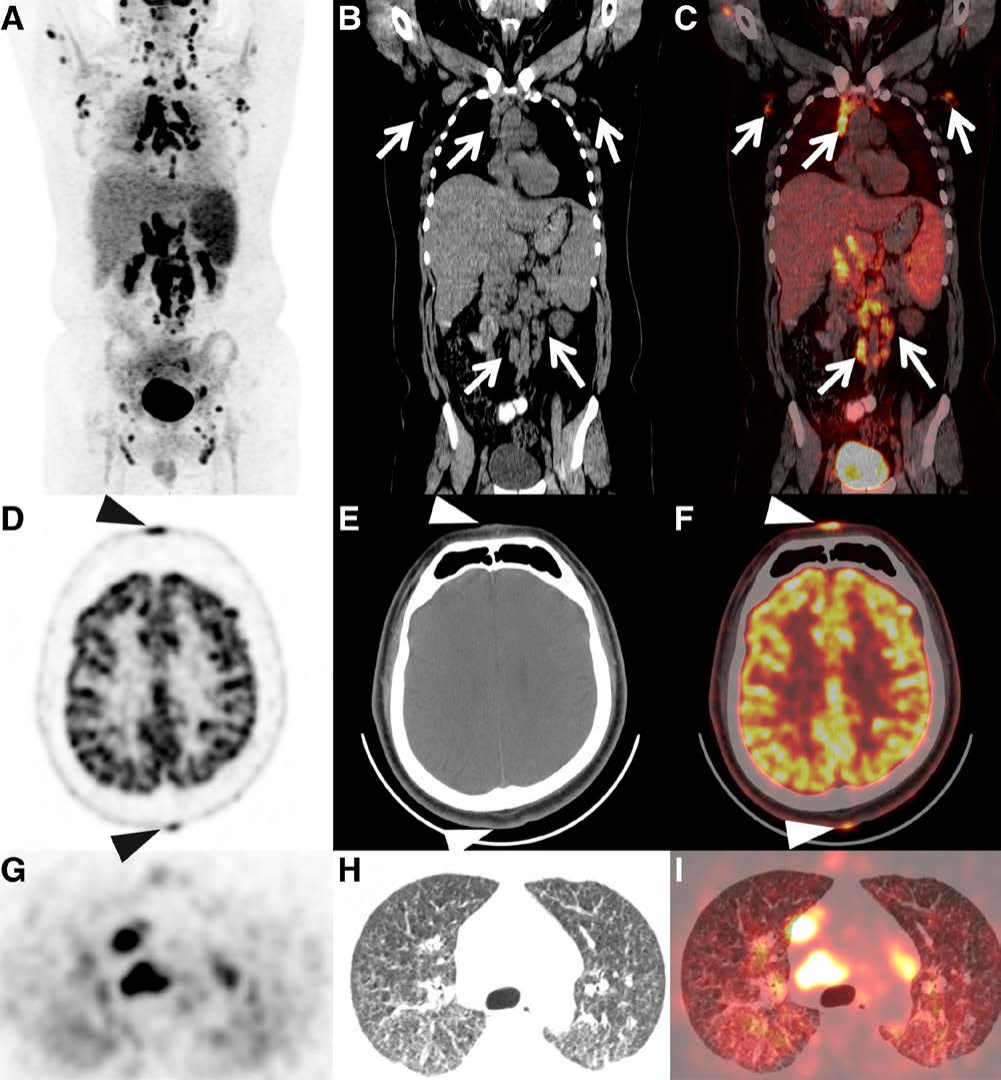
MIP image (A) showed multiple hypermetabolic lymph nodes in the upper and lower of diaphragmatic region. On coronal views of CT (arrows, B) and fused PET/CT (arrows, C), multiple hypermetabolic lymph nodes were revealed in the bilateral axillary, mediastinum, retroperitoneum, and adjacent to the bilateral iliac vessels with the SUV max ranged from 13.2 to 19.3. In addition, the metabolism of the spleen was diffusely increased. Multiple hypermetabolic nodules scattered on the scalp and face (arrowhead, D: PET, E: CT, F: fused PET/CT), and hypermetabolic micronodules were distributed diffusely in both lungs (G: PET, H: CT, I: fused PET/CT). CT = computed tomography, MIP = Maximal Intensity Projection, PET = Positron Emission Tomography, SUV = Standardized Uptake Value.

Ultrasonography showed diffuse enlarged lymph nodes in the neck, abdomen, and pelvis, which were suspected to be lymphomas. ^18^F- Fluorodeoxyglucose (FDG) Positron Emission Tomography (PET)/CT was performed to determine the cause of fever and lymph node enlargement. PET/CT imaging revealed multiple hypermetabolic lymph nodes in the upper and lower parts of the diaphragmatic region (Fig. 2A–C). In addition, metabolism in the spleen increased diffusely. Multiple hypermetabolic nodules were scattered on the scalp and face (Fig. 2D–F), and hypermetabolic micronodules were distributed diffusely in both lungs (Supplemental Digital Content [FIGURE2 G-I]). Based on the PET/CT results, malignant lymphoma was diagnosed, especially T-cell lymphoma involving the skin. Cryptococcal infection was diagnosed on the basis of blood culture and biopsy of the left inguinal lymph nodes. Periodic acid-Schiff staining was positive and acid-fast staining was negative. Laboratory testing revealed that the amount of cryptococcal capsular antigen in the serum was more than 100ug/L. In addition, *C. neoformans* was observed in cultures of secretions from rashes on the head and face. Finally, disseminated cryptococcosis was diagnosed.

**Figure 3.**
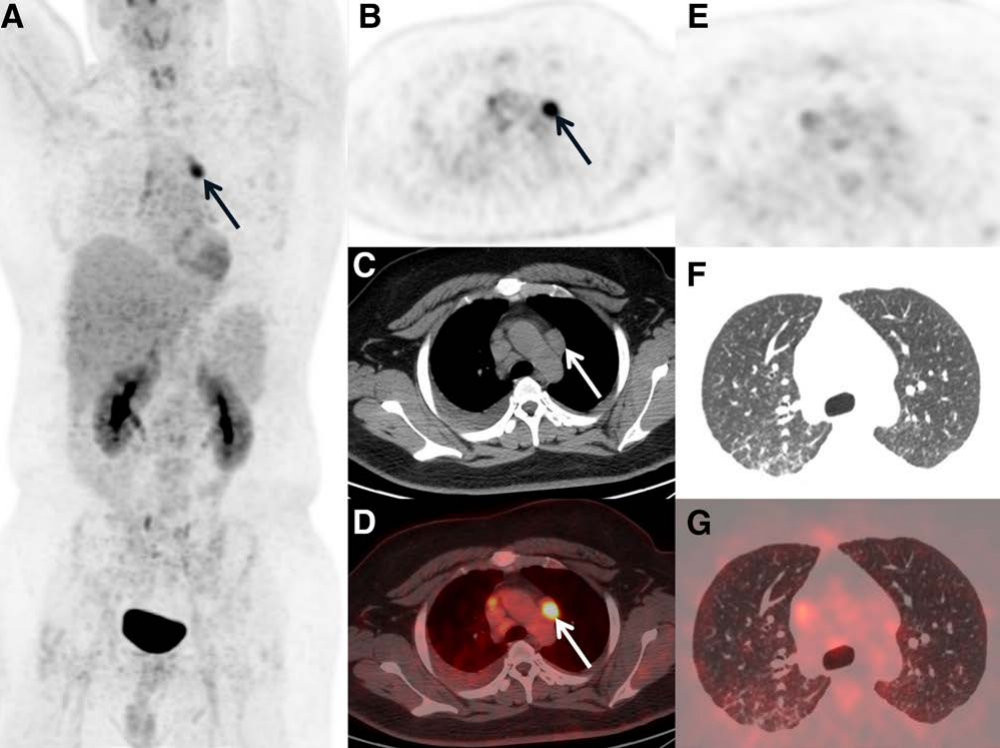
Only a few hypermetabolic lymph nodes and nodules remained existing in the mediastinum (arrows, A: MIP, B: PET, C: CT, D: fused PET/CT) and lung (E: PET, F: CT, G: fused PET/CT) after two months anticryptococcal therapy. Besides the metabolism of spleen back to normal. CT = computed tomography, MIP = Maximal Intensity Projection, PET = Positron Emission Tomography.

The body temperature returned to normal after two months of treatment with amphotericin B and fluconazole, and half a month after treatment with meropenem. Most of the hypermetabolic lymph nodes disappeared, which was confirmed by PET/CT reexamination, and only a few hypermetabolic lymph nodes and nodules remained in the mediastinum (Fig. 3A–D) and lung (Fig. 3E–G). In addition, the spleen metabolism returned to normal. The amount of cryptococcal capsular antigen declined to 9.52ug/L. According to the results of PET/CT and the reduction in cryptococcal capsular antigen, we considered that the condition improved after treatment.

## 3. Discussion

Disseminated cryptococcosis often occurs in immunocompromised patients and healthy individuals.^[3]^ Infection generally begins with the inhalation of *C. neoformans* or *C. gattii* which are fungal spores that spread hematogenously to other organs.^[3,4]^ *C. neoformans*, one of the most common pathogenic cryptococcal species, primarily infects immunocompromised hosts, whereas *C. gattii* mainly infects immunocompetent individuals. *C. gattii* is an encapsulated yeast that has traditionally been found in tropical and subtropical regions. Outbreaks have been recorded in temperate regions such as the Pacific Northwest of the United States,^[5,6]^ but there have been few reported cases of *C. gattii* in China.^[7]^ *C. neoformans* is found worldwide. The annual incidence of *C. neoformans* infection in immunocompetent hosts is approximately 0.4/1,00,000 to 0.9/1,00,000, while the annual incidence in immunocompromised patients, especially HIV-infected patients, is approximately 6%–10%.^[1]^

The central nervous system, lungs, and skin are the organs most vulnerable to *Cryptococcus*, which causes pulmonary infections, meningitis, and rashes.^[8]^ Cutaneous manifestations are observed in 10%–15% of cases. Cryptococcal infections of the skin are divided into 2 types: primary and secondary, with secondary infections being more common. Secondary infection suggests the presence of disseminated *Cryptococcus*, which is usually transmitted by the blood group. Cryptococcal infections of the skin mainly manifest as nodules, pustules, ulcers, and local cellulitis. These rashes are distributed throughout the body, mainly on the face and limbs. Lung involvement typically involves granulomatous reactions and infiltrates.^[9]^ Pulmonary cryptococcosis in adults and children often presents with symptoms, such as cough, fever, chest pain, and hemoptysis. Cryptococcal meningitis is characterized by fever, progressive headache, confusion, lethargy, irritability, disorientation, and marked behavioral changes. After cryptococcal infection, the symptoms of intracranial hypertension are often obvious, and there may be discomfort, such as headache, vomiting, and convulsions. Physical examination revealed a meningeal irritation.

Systemic disseminated cryptococcosis can be diagnosed in patients with cryptococcosis who present with infection at two discrete sites at the same time.^[10]^ Pathological diagnosis, etiological smear, and culture are the gold standards for diagnosis. It is usually obtained from the lesion, such as percutaneous lung biopsy specimens and local skin puncture specimens. A positive serum *Cryptococcus* capsular polysaccharide antigen latex agglutination test and positive sputum or bronchoalveolar lavage fluid smear can have clinical diagnostic value combined with medical history.

There are few reports on the clinicopathological features of cryptococcal lymphadenitis,^[11,12]^ which showed epithelioid granulomatous lymphadenitis with central necrosis of the granuloma and visible cryptococcal infection in the granuloma. It is difficult to distinguish this infectious lesion from a malignant tumor especially lymphoma by ^18^F-FDG PET/CT because of its extremely high metabolic features. However, PET/CT may be useful to assess the involvement of disseminated cryptococcosis and to evaluate residual disease after standard treatment. Furthermore, ^18^F-FDG PET/CT also plays a role in guiding biopsy when necrosis occurs after therapy^[13,14]^

## 4. Conclusions

Disseminated cryptococcosis, especially with lymph node involvement, is extremely rare and easily misdiagnosed as malignant lymphoma. ^18^F-FDG PET/CT may be useful for assessing the involvement of disseminated cryptococcosis and evaluating treatment effects.

## Author contributions

**Conceptualization:** Xinchao Zhang, Yanzhu Bian.

**Supervision:** Yanzhu Bian.

**Writing ‐ original draft:** Xinchao Zhang, Yanzhu Bian.
